# HERITABILITY OF AND EARLY ENVIRONMENT EFFECTS ON VARIATION IN MATING PREFERENCES

**DOI:** 10.1111/j.1558-5646.2009.00890.x

**Published:** 2010-04

**Authors:** Holger Schielzeth, Elisabeth Bolund, Wolfgang Forstmeier

**Affiliations:** 1 Max Planck Institute for OrnithologyEberhard-Gwinner-Str., 82319 Seewiesen, Germany; 2 E-mail: schielz@orn.mpg.de

**Keywords:** Heritability, mate choice, preference functions, quantitative genetics, sexual imprinting, zebra finch

## Abstract

Many species show substantial between-individual variation in mating preferences, but studying the causes of such variation remains a challenge. For example, the relative importance of heritable variation versus shared early environment effects (like sexual imprinting) on mating preferences has never been quantified in a population of animals. Here, we estimate the heritability of and early rearing effects on mate choice decisions in zebra finches based on the similarity of choices between pairs of genetic sisters raised apart and pairs of unrelated foster sisters. We found a low and nonsignificant heritability of preferences and no significant shared early rearing effects. A literature review shows that a low heritability of preferences is rather typical, whereas empirical tests for the relevance of sexual imprinting within populations are currently limited to very few studies. Although effects on preference functions (i.e., which male to prefer) were weak, we found strong individual consistency in choice behavior and part of this variation was heritable. It seems likely that variation in choice behavior (choosiness, responsiveness, sampling behavior) would produce patterns of nonrandom mating and this might be the more important source of between-individual differences in mating patterns.

Mate choice is a driving force of sexual selection and the origin of mating preferences is therefore an important issue for our understanding of evolution (Andersson and Simmons 2006). It is particularly challenging to quantify the sources of variation that produce between-individual differences within populations (Jennions and Petrie 1997; Widemo and Sæther 1999). The lifelong effects that would produce such variation are genetic differences between individuals and early rearing effects (Jennions and Petrie 1997; Widemo and Sæther 1999). Estimates of the genetic contribution to variation in mating preferences are particularly desirable, because heritability of preferences is a critical assumption of runaway selection (Fisher 1930; Lande 1981), and also other theoretical models of mate choice like indicator models (Fisher 1930; Zahavi 1975) or sensory bias models (Ryan 1998) would predict heritability of preferences. Thus, quantifying the relative contribution of genetic and early environmental effects on mating preferences will help in improving theoretical models of sexual selection (Widemo and Sæther 1999).

Mating preferences can be conceptually separated into preference functions, that is, the ranking order of stimuli, and choosiness, that is, the investment into mating with the preferred stimulus (Jennions and Petrie 1997; Widemo and Sæther 1999; Brooks and Endler 2001). Although both components contribute to the origin of mating biases and are hence both relevant for sexual selection, we will focus on preference functions, which can be considered mating preferences in the strict sense. We will use the term not only in the sense of preference functions for specific traits, but also in the general and abstract sense of ranking of stimuli without referring to specific traits. Note that this use is in agreement with the definition given by Jennions and Petrie (1997).

Substantial support for a genetic basis of preference functions comes from between-population differences in preferences and preferences for dichotomous traits (Majerus et al. 1982; Houde and Endler 1990; Bakker and Pomiankowski 1995; Velthuis et al. 2005). However, it is not always clear if such between-population genetic differences also explain variation of preferences within populations (Chenoweth and Blows 2006). Understanding the within-population variation in preferences (see Jennions and Petrie 1997 for a review) is important to understand the microevolutionary processes that take place within populations. Within populations, there is very good evidence for heritable variation in choosiness (Collins and Cardé 1990; Bakker 1993; Bakker and Pomiankowski 1995; Brooks and Endler 2001; Brooks 2002; Rodríguez and Greenfield 2003). Although this might result in mating biases and is thus relevant for mate choice and sexual selection, this does not mean that there is significant heritable variation in preference functions themselves.

Indeed, the number of studies addressing the within-population genetic basis of preference functions is very limited. Evidence for heritable variation comes from selection lines in stalk-eyed flies and in guppies (Wilkinson and Reillo 1994; Brooks and Couldridge 1999). Other studies have tested for genetic effects on preferences, some of them have found heritable variation (Moore 1989; Charalambous et al. 1994; Houde 1994), whereas others did not find significant heritabilities (Johnson et al. 1993; Breden and Hornaday 1994; Ritchie et al. 2005). These studies, however, do not quantify the amount of heritable variation. As far as we are aware (after a careful literature search including a reexamination of the studies presented in Bakker and Pomiankowski (1995) and a forward search for this seminal paper), there are only seven studies that present heritability estimates (Table 1): Three of them report significant heritabilities (point estimates for *h*^2^ between 0.14 and 0.51), whereas the others were nonsignificant and mostly very low.

**Table 1:** Published studies that present within-population heritability estimates of preference functions. We included only studies that analyze preference functions for traits that vary continuously within a population (although often only preferences for extremes were tested) and that estimate the heritability for the discrimination of mating stimuli (excluding estimates for the strength of a response to stimuli without discrimination).

Study	Species	Preference for	Choice method	Manipulation of preferred trait	Heritability (± SE)	Remarks
Collins and Cardé (1989)	Pink bollworm	pheromone composition (three component ratios)	sequential	yes	0.14 ±0.05	
Jang and Greenfield (2000)	Moth	pulse rate and asynchrony interval of calls	simultanuous	yes	0.21 ±0.13	
Iyengar et al. (2002)	Arctiid moth	body size	simultanuous	samples of defined differences	0.51 ±0.11	sex-chromosome linked inheritance
Gray and Cade (1999)	Field cricket	pulses per trill in calls	simultanuous	yes	0.34 ±0.17	
Simmons (2004)	Field cricket	long chirp relative to short-chirp song elements	simultanuous	yes	<0.00	
Hall et al. (2004)	Guppy	male attractiveness (as measured in choice chamber)	simultanuous	samples including extremes	−0.07 ±0.13	selection lines (both direct and indirect selection)
Brooks and Endler (2001)	Guppy	coloration & size (several measures), max for brightness contrast	simultanuous	no	0.10 ±0.11	maximal heritability from a large range of tests

Environmental processes that act during early life and that have the potential to produce between-individual differences in preferences have also not been fully studied. Sexual imprinting is such a process of vertical transmission of preferences that would result in similar preferences within and potentially different preferences among broods or litters. It involves the formation of preferences early in life usually by imprinting on the parental phenotypes (Immelmann 1975). There is a very good evidence for sexual imprinting on heterospecific foster parents, morphs, or novel ornaments (e.g., Immelmann 1975; ten Cate and Bateson 1988; Qvarnström et al. 2004; Burley 2006). The evidence for sexual imprinting on continuous variation within a single population (and thus not involving the categorization of individuals into distinct classes) is limited and ambiguous (Bereczkei et al. 2004; Schielzeth et al. 2008). Hence, we currently do not know if sexual imprinting is involved in producing within-population variation in preference. There are family effects other than sexual imprinting that might affect mating preferences. For example, early rearing conditions might influence mating preferences, although this is more likely to affect choosiness rather than preference functions.

Most studies, in particular those on the heritable variation of preference functions, have focused on specific traits and have used manipulation (Collins and Cardé 1989; Gray and Cade 1999; Jang and Greenfield 2000; Simmons 2004; Ritchie et al. 2005) or have chosen extreme phenotypes (Houde 1994; Wilkinson and Reillo 1994; Brooks and Couldridge 1999; Hall et al. 2004) to increase the variance along a specific axis of ornamentation. This is a valuable approach, but does not allow an understanding of sources of variation in preferences for potential mates in their full multidimensionality (Candolin 2003; Fawcett and Johnstone 2003), because it creates dichotomous groups. Hence, we have employed an experimental design that allows testing for the similarity in preferences between (1) genetic sisters and (2) foster sisters in a population of zebra finches when presented with the natural range of between-male variation within a population. A full individual cross-fostering scheme enabled us to disentangle genetic and early rearing effects. For the first time in a population of animals, we quantify the genetic and early rearing effects on mating preferences simultaneously. We focus on preference functions (in the abstract sense of ranking stimulus individuals without a focus on specific traits), but at the same time present results on between-individual variation in choice behavior.

## Methods

### SUBJECTS AND HOUSING

We used 176 female and 176 male zebra finches *Taeniopygia guttata castanotis* from a large captive population (for details on housing see Bolund et al. 2007). All individuals belong to a single generation, but were bred in two cohorts (September to November 2005 and April to June 2006). All individuals had been cross-fostered individually within 24 h after egg-laying, which ensured that all broods consisted of only unrelated chicks and that all subjects were raised by foster parents unrelated to all nestlings (Schielzeth et al. 2008). Genetic parentage of chicks was ascertained by genotyping chicks for 10 polymorphic microsatellite markers (Forstmeier et al. 2007) and subsequent parentage assignment by exclusion. Brood size varied between one and six (mean ± SD: 3.4 ± 1.1).

Juveniles were separated from their foster parents at 35 days of age and were kept in juvenile peer groups until an age of about 100 days (47% in unisexual peer groups, 53% in mixed-sex peer groups). Peer groups varied in size between four and 36 individuals (mean ± SD: 16.7 ± 13.7). The majority of foster sisters (84%) and genetic sisters were kept in different peer groups between day 35 and day 100. Variation in peer groups size and composition was introduced for reasons that are beyond the scope of this study. It follows from these rearing conditions that (1) genetic sisters had never been kept together up to (at least) day 100 and a similarity between them would arise only for genetic reasons or maternal effects and (2) most fosters sisters largely shared only the rearing environment between day 0 and 35 and hence, a similarity between them would arise almost exclusively from shared conditions during this early period.

Subjects were sexually mature at the time of testing (birds from 2005: 557 ± 21 days, mean ± SD, birds from 2006: 339 ± 21 days). Throughout the trial period, birds were housed in doublets of same-sexed individuals (but not with their genetic or foster sister).

### EXPERIMENTAL DESIGN

We tested mating preferences of 44 pairs of genetic full-sibling sisters that were raised apart (all from different families) and 44 pairs of unrelated foster sisters that were reared together in the same brood (except for one pair, in which foster sisters were raised in different broods but by the same foster pair). Hence, foster sisters shared the same rearing environment (same brood or at least same foster parents) up to day 35, when they were randomly assigned to peer groups (see above).

Each female had eight two-way choice trials. As stimulus birds we used a total of 176 males that were randomly assigned to duplets of one focal male and one opponent male. We ensured that they were unrelated and unfamiliar to all females they were tested with. Focal males were always tested with the same opponent male. This was done to remove interaction effects between stimulus males (particular males might appear more attractive when presented with one opponent male than when presented with another, e.g., Bateson and Healy 2005). Stimulus males from the same duplet were not allowed to interact with each other either during trails or between trials.

Male duplets were used as stimulus birds for four pairs of genetic sisters (eight females) and four pairs of foster sisters (eight females). These were tested in two blocks: two pairs of genetic sisters and two pairs of foster sisters first and then another two pairs of genetic sisters and two pairs of foster sisters. Hence, male pairs were used exactly 16 times and each female had exactly eight trials. This amounted to 1408 choice chamber trials. Females had one trial per day and the eight trials were run on four consecutive days (first four trials), followed by one day of break and another four trails on four consecutive days (trials five to eight). The sequence of testing the females with male pairs was randomized within blocks. Male pairs were always tested in the same of eight identical choice chambers, but the sides of the stimulus cages were randomly assigned to the two males.

Because within blocks two pairs of genetic sisters and two pairs of foster sisters were tested with the same male, we were able to calculate the similarity between genetic sisters and foster sisters and could compare this to the similarity between unrelated females (we use the term in the sense of unrelated and not sharing the same foster parents). We chose to compare only unrelated pairs within blocks, because the order of testing was randomized within blocks and hence, genetic and foster sister pairs were not tested closer in time than pairs of unrelated females. Every female was involved in three pairs of comparisons with unrelated females (Table 2). This means that within each block of eight females, we formed 12 pairs of unrelated females in the analysis (264 pairs of unrelated females in total). We could have formed more pairs of unrelated females (excluding cage mates during the testing period we could have formed 20 such pairs per block), but we consider this unnecessary, because the number of unrelated female comparisons is already six times as large as the number of genetic sister and foster sister comparisons, respectively. The informative comparisons are limited by the number of genetic sister and foster sister pairs, so that more pairs of unrelated females would not improve the estimates significantly.

**Table 2:** Illustration of the experimental design. Two pairs of genetic sisters (here A_1_–A_2_ and B_1_–B_2_) and two pairs of foster sisters (here C1–C2 and D1–D2) were tested with eight sets of males. Between trials, they were housed in duplets of two females per cage (C). We compared the agreement between genetic sisters (GS) and foster sisters (FS) to the agreement between unrelated females that did not share the same foster parents (U).

Individual	A_1_	A_2_	B_1_	B_2_	C_1_	C_2_	D_1_	D_2_
A_1_	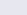							
A_2_	GS	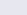						
B_1_		U	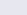					
B_2_	U	C	GS	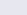				
C_1_		U	C	U	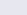			
C_2_	U		U		FS	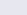		
D_1_	C	U		U		U	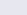	
D_2_	U		U		U	C	FS	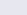

### CHOICE CHAMBER TRIALS

We used a two-way choice chamber setup identical to the one described in Schielzeth et al. (2008) except for two changes. First, the compartment of the accompanying female (as described in Schielzeth et al. 2008) was empty and inaccessible to the choosing female. Second, the compartments close to the male stimulus cages were equipped with two parallel perches to allow the female ritualized hopping. Females could see males even from the central compartment. They typically started approaching one of the males shortly after trials had started and in most trials (90%) females visited each male at least once.

Presence in all three compartments (two close to the stimulus males at either end and a neutral zone in the middle) was recorded automatically using infrared sensors and photoelectric relays (Schielzeth et al. 2008). We used eight identical choice chambers that allowed us running eight trials simultaneously. During trials, subjects had no visual contact to any individual that was not involved in the trial. Trials lasted 1 h.

As described in Schielzeth et al. (2008), we calculated the proportion of time spent with the focal male (time spent in the compartment close to the focal male divided by the sum of the times spent in the compartments close to either of the two males) and used this as a measure of preferences. Time spent with males has been shown to correlate with sexual preferences (Witte 2006; Forstmeier 2007) and shows moderate, but significant repeatabilities when measured several weeks apart (0.26–0.29, Forstmeier and Birkhead 2004; Schielzeth et al. 2008).

We also analyzed the similarity in dichotomized preferences for individual males by referring to the male a female spent most time with as the preferred male. Although in the extreme case a single second of difference in time allocation might decide which male was the preferred one, this analysis is important to completely disentangle preference functions and choice behavior as some females might generally distribute their time more evenly than others. We also conducted analyses, in which we limited the dataset to trials in which females showed clear or very clear preferences (i.e., they spent at least 70% or 85% of their time with one male). This substantially reduces the number of data points, makes the design unbalanced, and limits the analysis to a subset of females in the population (the ones that distribute their time less evenly), but measurement error is potentially reduced when only clear decisions are included.

Although the preference function was analyzed as the proportion of time spent with an individual male, we calculated four aspects of choice behavior that are not directly related to preference functions: (1) the total number of registrations from the motion-sensitive sensors as a measure of hopping activity, (2) the number of transitions from one outer compartment to the other as a measure of the number of comparisons between stimulus males, (3) the total proportion of time spent close to any one of the males (i.e., not in the neutral zone) as a measure of female interest in males, and (4) the absolute deviation of time allocation between males from 0.5 (no discrimination) as a measure of clarity of the choice.

### DATA ANALYSIS

We used angular transformation of percent time spent with the focal male (*y*’= arcsine(√*y*)) for all analyses. The number of transitions between males was log-transformed (*y*’= ln(*y*+ 1)). The percent time close to any male was transformed as *y*’= y^5^. These transformations were applied to achieve better fit to normal distributions. We calculated the repeatability of male attractiveness as the variance component of focal male identity on time allocation to the focal male (assessed by eight females) in a random-intercept model. In the absence of fixed factor predictors, variance components are the proportion of variance explained by a random-intercept effect relative to the total variance. We used likelihood-ratio tests to test for the significance of variance components.

To estimate the heritability of and early environment effects on preferences, we calculated the correlation between the two females forming one pair (genetic sisters or foster sisters) assessing the same eight sets of males. We used the mean and the standard error of the population of correlation coefficients (one per pair of females) as an effect size estimate (for either the genetic or the shared early environment effect on preferences). However, because there was some overall agreement between females on male attractiveness (see results) these estimates include effects of between-female agreement independent of relatedness and shared foster environment effects. To control for this, we calculated the partial correlation coefficients for each sister pair while controlling for the mean preference of the other 14 females that were tested with the same set of males. We then calculated the correlation across all pairs of sisters as described above. The population estimate for the correlation represents half the heritability of female preference functions (because in full-siblings 50% of the alleles are identical by decent) in the analysis of genetic sisters, but the entire shared environment component in the analysis of foster sisters. The resulting estimate of heritability includes possible maternal effects and parts of dominance variance and epistatic interactions, but is independent of the early environment (as ensured by cross-fostering).

We used variance component analyses to analyze the heritability of and early rearing effects on female behavior in the choice chamber (female activity, number of comparisons, clarity of the choice, time spent with males). Models included the trait under consideration as a response and female identity, genetic pair identity, foster pair identity, male pair identity, and choice chamber identity as random-intercept effects. Genetic pair identities were coded the same for each pair of genetic sisters, whereas unrelated foster sisters were coded with unique genetic pair identities. Similarly, foster pair identities were unique for females from genetic sister, but identical for each pair of females from foster sisters pairs. The variance component of genetic pair identity represents the within-full-sibling repeatability. This is half the heritability of the respective trait (see above). The variance component of foster pair identity represents the full-shared early environment effect.

All calculations were done in R 2.8.0 (R Development Core Team 2008). We used the lmer function from the lme4 package (Bates et al. 2008) for variance component analyses.

## Results

### PREFERENCE FUNCTIONS

**Figure 1:**
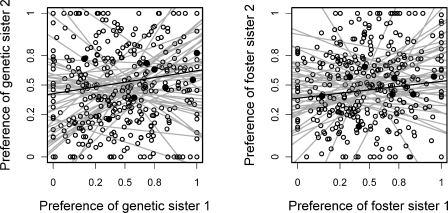
Similarity in preferences between pairs of genetic sisters and pairs of foster sisters. The preferences were measured as the proportion of time spent with the focal male in a two-way choice chamber. They were normalized by angular transformation (*y*’= arcsine(√*y*)) for display and analysis, but percentage-scale labels are shown in the plots. Forty-four pairs of genetic sisters and 44 pairs of foster sisters were tested with eight sets of two males each. Regression lines are shown for each pair of sisters. The black data points and the solid black regression line highlight a typical example (one close to the population mean) for the eight pairs of trials of one pair of sisters.

The repeatability of male attractiveness was low but significant (variance component for male identity: 0.103 ± 0.001, LRT: χ^2^_1_= 63.4, *P* < 10^−14^). The correlation in preferences between genetic sisters was low overall (*r*= 0.12 ± 0.07, *n*= 44, *P*= 0.087, Fig. 1), and even lower when controlling for between-female agreements on male attractiveness measured as the mean attractiveness as judged from the other 14 females that were tested with the same set of males (*r*= 0.05 ± 0.07, *n*= 44, *P*= 0.42). This results in a broad-sense heritability estimate for female mating preferences of *H*^2^= 0.10 ± 0.14 (including maternal effects, parts of dominance and epistatic interactions, but no early rearing effects). The correlation in preferences between unrelated foster sisters was low (*r*= 0.12 ± 0.06, *n*= 44, *P*= 0.051, Fig. 1), and even lower when controlling for between-female agreement on male attractiveness (*r*= 0.05 ± 0.07, *n*= 44, *P*= 0.52).

**Figure 2:**
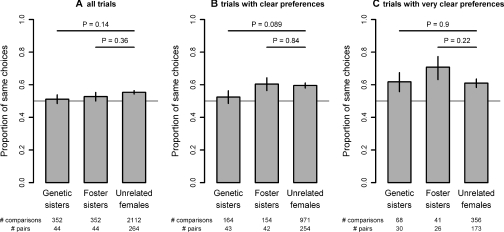
Between-female agreement in mate choices in a two-way choice chamber. Each female had eight trials and proportion of agreements in dichotomized preferences (identity of the male that a female spent the larger fraction of time with) was calculated between pairs of genetic sisters, pairs of foster sisters and pairs of unrelated females. The three plots show all trials (A), only trials with time allocation to the preferred male of >70% (B), and only trials with time allocation to the preferred male of >85% (C). Because limiting the comparisons to only clear choices means excluding trials with less clear choices, sample sizes vary among plots. Differences between the agreement among genetic sisters and the agreement among foster sisters relative to the agreement among unrelated females were tested in a generalized linear model (GLM) with binomial error structure and logit link and a single categorical predictor (type of female pair) with three levels. *P*-values refer to the contrasts between the two types of females and the reference category (unrelated females).

We also analyzed the agreement in dichotomized preferences between genetic sisters and foster sisters. Little more than 50% of all trials showed an agreement between females, and neither genetic sisters nor foster sisters differed from unrelated females (Fig. 2). When limiting the data to comparisons in which both females spent more than 70% or more than 85% of their time with the preferred male, the agreement becomes larger, but genetic sisters and foster sisters still did not differ from unrelated females. In the full sample and in the two subsets, the agreement between foster sisters was slightly larger than between genetic sisters, although these differences were nonsignificant.

### CHOICE CHAMBER BEHAVIOR

We found significant within-female repeatability of time spent close to males, the number of comparisons between males, female activity, and clarity of choice (total female effect in Table 3). All these traits had small male pair identity and choice chamber identity effects, although some were significant (Table 3). All traits showed some indication of genetic effects as measured by the similarity between genetic sisters (Genetic component in Table 3), but no indication of foster environment effect as measured by the similarity between foster sisters (Early rearing environment in Table 3). Because the broad-sense heritability is twice the intraclass correlation coefficient for full-siblings, this results in estimated variance components of *H*^2^= 0.10–0.30 (including maternal effects, parts of dominance and epistatic interactions, but no early rearing effects). All traits showed a large degree of between-female variation in behavior after controlling for genetic and foster environment effects (Residual in Table 3), which indicates other permanent environmental effects not shared between foster sisters.

**Table 3:** Variance component (VC) analysis of female behavior in the choice chamber. Likelihood-ratio tests were used for significance testing. There were 176 females (44 pairs of genetic sisters and 44 pairs of foster sisters) that had eight trials each, 88 sets of males that had 16 trials each, and eight choice chambers with 176 trials in each. The broad-sense heritability is twice the similarity between full-siblings (this estimate includes possible maternal effects and part of dominance and epistatic interactions). The total female effect is the sum of genetic, foster environment, and additional female identity effects.

	Clarity of choice			Time with males			Number of comparisons			Female activity		
	VC	χ^2^	*P*	VC	χ^2^	*P*	VC	χ^2^	*P*	VC	χ^2^	*P*
Genetic component	0.05	1.36	0.51	0.06	17.4	<10^−3^	0.12	19.2	<10^−4^	0.15	16.8	<10^−3^
Early rearing environment	0.00	0.00	1.00	0.00	0.0	1.00	0.00	0.00	1.00	0.00	0.0	0.99
Additional female identity effects	0.18	13.5	<10^−3^	0.43	22.9	<10^−5^	0.45	20.5	<10^−5^	0.35	19.5	<10^−4^
Male pair component	0.00	0.19	0.66	0.00	0.00	1.00	0.02	13.7	<10^−3^	0.03	25.7	<10^−6^
Choice chamber component	0.00	0.00	0.98	0.01	16.8	<10^−4^	0.02	17.4	<10^−4^	0.03	13.7	<10^−3^
Residual	0.77			0.50			0.39			0.44		
Broad-sense heritability	0.10			0.12			0.24			0.30		
Total female effect	0.23			0.50			0.57			0.50		

## Discussion

We measured the similarity in preferences and choice behavior between genetic sisters and foster sisters for a population of female zebra finches in a two-way choice chamber. In accordance with earlier work (Forstmeier and Birkhead 2004), the overall agreement between females on male attractiveness was low. The between-female agreement was slightly higher, when including only trials with very clear preferences than when including all trials (61% vs. 55%, Fig. 2). Whether we included all trials or only trials in which females showed very clear preferences or not, the main conclusions remained the same: Genetic sisters and foster sisters did not show higher agreement than unrelated females.

Our results indicate that heritability of and early rearing effects on preference functions are very low. When analyzing the strength of the preferences as in Figure 1 (a mixture of preference functions and strength of choice), the broad-sense heritability was low and nonsignificant (*H*^2^= 0.10 ± 0.14). At the same time, we found very consistent choice behavior of individual females in the choice chamber. Part of this between-individual variation in behavior was heritable (point estimates for *H*^2^ between 0.10 and 0.30), whereas the shared environment component was estimated to zero for all traits.

Seven published studies have estimated within-population heritability of preference functions for continuous variation, usually by measuring the relative time spent with males or the proportion of visits (Table 1). Our estimate of the heritability based on the proportion of time spent with males (0.10) is very close to the median of these estimates (0.14). However, the relative time allocation used in our study and some other studies might include aspects of choice behavior and thus might produce a somewhat higher heritability estimate as compared to pure preference functions. In our data, there was no evidence for a similarity in the dichotomized outcome of choices between genetic sisters. Because the agreement between genetic sisters was even slightly lower than between unrelated females, this indicates that the heritability of preference functions was indeed very close to zero. It is hard to imagine processes that would make genetic sisters dissimilar in their preferences; hence the negative estimate is likely to be due to sampling variance alone.

Although not significantly different from zero in our and several published studies (Table 1 and references in the introduction), we do not think that the heritability of preference functions is actually zero. The evidence from selection lines and quantitative genetics in insects and fish (Wilkinson and Reillo 1994; Brooks and Couldridge 1999) as well as between-population differences (e.g., Velthuis et al. 2005) give convincing evidence for nonzero heritabilities. However, the within-population heritable variation appears to be very low in most studies.

Brooks and Endler (2001) estimated the heritability of preference functions for a large number of traits in guppies. All of them were nonsignificant and mostly very low (max. *h*^2^= 0.11). However, they found significant heritability of responsiveness, that is, a specific aspect of choosiness (*h*^2^= 0.27 ± 0.13) and conclude that heritable variation in responsiveness might mask variation in preference functions and may be the most relevant source of between-individual variation in mating preferences. Our results support this suggestion, because we find highly repeatable and also heritable variation of choice behavior. Although it is not clear, how the specific behaviors translate into mating behavior in the wild, this finding might relate to differences in mate sampling. The high within-female repeatability clearly indicates individuality in choice behavior.

Beside the potential for masking variation in preference functions, variation in choice behavior might also be confused with variation in preference functions (Wagner 1998). For example, female sticklebacks show a preference for redder males, but there is heritable variation in the strength of discrimination (Bakker 1993). Females that show strong preferences had brothers with redder coloration compared to females that do not discriminate (Bakker 1993). Because the strength of preferences is an aspect of choosiness, this could potentially be explained by condition dependence in choosiness (Burley and Foster 2006). If condition is heritable and is also expressed in males by showing larger areas of red coloration, this can produce an ostensible genetic correlation between a trait and a preference for this trait.

Our preference tests also allowed a strong test for sexual imprinting effects on mating preferences. Early rearing effects as estimated from time allocation and between-foster sister agreement on attractiveness were very low and nonsignificant. There was some indication that the agreement between foster sisters increased when limiting the data to only very clear decisions, although this effect was clearly not significantly different from the agreement between unrelated females (*P*= 0.22). This very low and clearly nonsignificant effect of shared early-rearing conditions on preference functions is in agreement with an earlier finding in our population (Schielzeth et al. 2008). The finding we present here was derived from an independent set of experiments and uses a different approach. Both studies, however, are limited to sexual imprinting (Schielzeth et al. 2008) or more generally shared early-rearing conditions (this study) during the early period of life (day 0–35). Day 35 is after nutritional independence and around the time when young zebra finches would typically start leaving their parents (Zann 1996). It is possible that preferences are formed later on during adolescence (e.g., in peer groups), but our studies show that the parents do not play an important role in the formation of mating preferences.

Hence, the only positive evidence for sexual imprinting on continuous variation to date stems from humans (Bereczkei et al. 2004). Given this lack of further evidence, we conclude that sexual imprinting is probably relevant for species recognition (e.g., Immelmann 1975; ten Cate and Bateson 1988; Qvarnström et al. 2004; Burley 2006) and for sex recognition (ten Cate et al. 2006), but does not seem to explain between-individual differences in mating preferences within a single population. However, future studies explicitly testing the within-population relevance of sexual imprinting will reveal if this conclusion is general or if sexual imprinting is an important proximate cause of within-population variation in preferences in other species.

We conclude that empirical support for early rearing effects on preferences is currently very limited and is unlikely to play an important role for variation in preferences within populations. There is more evidence for heritable variation of preference functions in published studies, although this is not always strictly separated from heritable variation of choosiness and responsiveness. Estimates are usually low and often nonsignificant (as in our study). Hence, heritable variation is apparently not strong enough to explain much of the between-individual differences in preference functions. In contrast to the weak effects on preference functions, we find strong evidence for individual and heritable components to choice behavior. This has the potential to be the most important source of nonrandom mating patterns by influencing realized choices independent of preference functions.
